# Hodgkin lymphoma transformation of chronic lymphocytic leukemia: cases report and discussion

**DOI:** 10.1007/s12032-013-0800-8

**Published:** 2013-12-15

**Authors:** Maciej Kaźmierczak, Renata Kroll-Balcerzak, Andrzej Balcerzak, Elżbieta Czechowska, Lidia Gil, Krzysztof Sawiński, Andrzej Szczepaniak, Mieczysław Komarnicki

**Affiliations:** 1 Department of Hematology and Bone Marrow Transplantation, Poznan University of Medical Sciences, Szamarzewskiego 84, 60-569 Poznan, Poland; 2 Department of Internal Medicine and Hematology, Stanisław Staszic Regional Specialist Hospital, Rydygiera 1, 64-920 Piła, Poland

**Keywords:** Hodgkin lymphoma, Chronic lymphocytic leukemia, Histological transformation, Trephine biopsy

## Abstract

B-cell chronic lymphocytic leukemia/small lymphocytic lymphoma (CLL/SLL) is the most common form of leukemia affecting adults in Europe and North America. Large B-cell lymphoma known as Richter’s syndrome (RS) may develop approximately in 3–15 % patients. Furthermore, other hematological malignancies may also occur as RS variants, among them—Hodgkin lymphoma (HL). CLL/SLL transformation into HL is observed in about 0.5 % of patients, and till now, fewer than 100 cases have been reported in the medical literature. We present two cases of HL transformation of CLL/SLL and review the previously published literature.

## Introduction

B-cell chronic lymphocytic leukemia/small lymphocytic lymphoma (CLL/SLL) is the most common form of leukemia affecting adults in Europe and North America. The clinical course of CLL/SLL is variable. In most cases, it is indolent and only fewer than one-third of patients represent an aggressive disease, requiring treatment [1]. In approximately 3–15 % patients with CLL/SLL, large B-cell lymphoma (LBCL) known as Richter’s syndrome (RS), may develop. Apart from that, other hematological malignancies such as prolymphocytic leukemia, hairy cell leukemia, Burkitt lymphoma, high-grade T-cell non-Hodgkin lymphoma (NHL), multiple myeloma, acute lymphoblastic leukemia, and Hodgkin lymphoma (HL) may also occur as RS variants [2]. Hodgkin transformation of CLL/SLL (sometimes called Hodgkin variant of Richter transformation or syndrome) is observed in around 0.5 % of patients. An increased risk (observed-to-expected ratio 7.69) of HL in this group of patients was established [3]. CLL/SLL patients are also at a significantly increased risk of developing a second malignant neoplasm such as cancers of lung, brain, eye, and malignant melanoma [3]. Herein, we report two patients with CLL/SLL who developed HL.

## Case reports

### Case 1

A 48-year-old woman was admitted to hospital in April 2010 because of leukocytosis (lymphocytosis), lymphadenopathy, and splenomegaly. She was diagnosed with CLL by morphology, flow cytometry, and histopathological evaluation of the excised cervical lymph node. The stage II according to Rai classification was established. In May 2010, chemotherapy with fludarabin and cyclophosphamide (FC) was introduced due to progressive disease (lymphocytosis). After five courses of therapy, the complete remission (RC) was established. RC lasted 14 months. In December 2011, progressive lymphadenopathy was observed. The active metabolic process in lymph nodes was confirmed by PET-CT. There was no evidence of residual CLL, and therefore, peripheral lymph node was examined for the second time. On the basis of histopathological evaluation of excised cervical lymph node, the diagnosis of classic HL, mixed cellularity (MC) type was established. Immunohistochemical stains revealed the Hodgkin and Reed–Sternberg cells (H–RS) to be positive for CD30 and CD15 and negative for CD3 and CD20. The pathological material was also investigated for latent Epstein–Barr virus (EBV) and was positive in staining for latent membrane protein 1 (LMP1) by immunohistochemistry and for EBV small nuclear RNA transcripts (EBER) by in situ hybridization. The stage IIIA (according to Ann Arbour classification) of HL was established, and the treatment with ABVD regimen (adriablastin, bleomycin, vinblastin, and dacarbazin) started in January 2012. After two courses of chemotherapy, PET-CT was performed. The patient was considered as high risk and qualified to autologous hematopoietic stem cell transplantation (autoHSCT). Due to resistance to the first-line therapy, the second-line therapy was introduced—three courses of DHAP (dexamethasone, cytarabine, and cisplatin). However, this therapy failed as well, and the patient was treated with IVE regimen (ifosfamide, epirubicin, and etoposide). After two courses of IVE, complete remission was achieved and stem cell collection was performed for the autoHSCT. Just before procedure, right axillary lymph node enlarged. However, we decided to proceed with autoHSCT, and after it, radiotherapy of the involved field was planned. Finally, autoHSCT was performed in January 2013, and cytopenia period was complicated by septic shock. Additionally, the patient was in very poor performance status due to toxic heart failure for several weeks after autoHSCT, so radiotherapy was not possible. Four months after transplantation, we observed progressive disease with lymphadenopathy (histopathological evaluation of excised lymph node revealed again MC HL), hepatosplenomegaly, and further worsening of the performance status. She was disqualified from intensive therapy, and palliative therapy was introduced. In July 2013, she died due to disseminated disease.

### Case 2

**Fig. 1:**
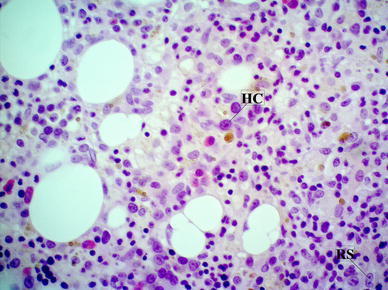
Hodgkin (*HC*) and Reed–Sternberg (*RS*) cells in bone marrow (trephine biopsy, hematoxylin and eosin staining, magnification ×400)

A 39-year-old man felt well until October 2010, when he presented massive lymphadenopathy, fever, and night sweats. He was subsequently diagnosed with CLL/SLL by histopathological evaluation of the excised cervical lymph node. Bone marrow revealed infiltration of CLL/SLL cells, but the criteria of CLL were not met (flow cytometry of blood demonstrated that only 0.06 g/L cells were CD19+CD5+). The diagnosis of non-Hodgkin lymphoma IVB (histologically SLL) was established. He completed four cycles of FCR (fludarabine, cyclophosphamide, and rituximab) from May to September 2011. He showed no response to chemotherapy, so the diagnostic procedure was repeated. A bone marrow biopsy showed three focal areas of fibrosis containing Hodgkin and Reed–Sternberg cells (Fig. 1). Immunohistochemical stains revealed the large cells to be positive for CD30 and CD15 and negative for CD45. For a definitive diagnosis, additional cervical lymph node biopsy was performed. The biopsy findings showed occurrence of classic HL (MC type), with no evidence of CLL/SLL. Immunohistochemical analysis revealed cells positive for CD30 and negative for CD45 and CD20. EBV LMP1 and EBER were positive in these cells. The diagnosis of Hodgkin lymphoma IVA was established in January 2012. The patient underwent treatment with two cycles of BEACOPP (bleomycin, etoposide, doxorubicin, cyclophosphamide, vincristine, procarbazine, and prednisone). Because of progression of the disease, the second-line therapy according to ESHAP schedule (etoposide, cytarabine, methylprednisolone, and cisplatin) was introduced. After one course, the resistance to the therapy was observed. He died in May 2012 because of multiple organ dysfunction syndrome (MODS).

## Discussion

The occurrence rate of HD in CLL/SLL patients has been reported to be around 0.5 %, but till now, only fewer than 100 such cases have been reported [3–5]. The pathomechanism of neoplasm evolution is not well elucidated. Two types of Hodgkin transformation of CLL/SLL have been described. Type 1 transformation is characterized by H–RS cells scattered in a background of CLL cells, which could suggest histological progression of the underlying CLL cells, especially when the H–RS cells express B-cell markers [6]. In type 2 transformation, the origin of H–RS is different from CLL cells because H–RS cells are present in a typical polymorphous, inflammatory background [7]. In this type, it is unclear whether HL represents a clonal transformation from CLL/SLL or is secondary to treatment, or it represents a de novo neoplasm [5]. In our patients, we observed type 2 transformation. In the first case, it occurred after 14 months of CR (achieved after FC therapy) of CLL. Till now, there have been only a few reports in the world literature on such patients [5, 8]. In the second case, transformation was connected with progression of the disease just after completing FCR treatment. In both cases, we observed presence of EBV LMP1, carrying a strong transforming and antiapoptotic potential, and EBER, a pattern characteristic of latency type II EBV infection [9]. EBV can be considered to represent a prototype of oncogenic viruses that behave as direct transforming agents. It may infect CLL cells, resulting in a nonpathogenic latent infection of memory B lymphocytes. The presence of EBV genomes and the constant expression of viral proteins in all neoplastic cells suggest that tumors develop from a single, EBV-infected cell and support its involvement in the pathogenesis of several EBV-related tumors, among them HL, and other tumors for which a causal role of EBV seems unlikely, for example, CLL/SLL [10, 11]. Additionally, a much higher association between the presence of EBV in the H–RS cells of the patients with HL transformation than in classical Richter’s transformation (88 vs. 16 %) could suggest pathogenic role of EBV in HL transformation of CLL [12]. Some authors have shown an increased frequency of EBV in patients previously treated with fludarabine, which took place also in our patients [13]. The prevalence of EBV in H–RS cells varies according to the histological subtype and epidemiologic factors. The highest frequency (about 75 %) is found in MC HL, and the lowest frequency is found in nodular sclerosis HL (10–40 %) [14]. Both of our patients presented MC type of HL.

There are no clearly established risk factors that could identify the subgroup of patients predestined to HL transformation of CLL. One of them could be immunosuppression associated with the biology of CLL/SLL or with the prior treatment [15]. It is of great concern that therapy may further increase the risk of transformation or of a second neoplasm. However, until now, there is no clear evidence that alkylating agents or purine analogues may be associated with an increased incidence of transformation in patients with CLL [16]. Bockorny et al. [13] showed that fludarabine prolonged the period of transformation from CLL to HL, but decreased the subsequent survival period after Hodgkin variant of Richter transformation occurred.

The majority of patients with HL transformed from CLL/SLL have high-risk features if prognostic models for de novo HL are applied [4]. This more aggressive course of this type HL did not seem to be related to either the Ann Arbor stage or the histological subtype on transformation [13]. Unfavorable prognosis was observed especially when active CLL/SLL continued at the time of transformation to HL [5]. The presence of EBV in the common NHL and HL confers a much worse prognosis after conventional therapy [10].

On the basis of the world medical literature, the HL transformation of CLL was observed mainly in older and male patients. B-symptoms, lymphadenopathy, and hepatosplenomegaly were the most common clinical manifestations at the time of transformation. The most common histological subtype was mixed cellularity, followed by nodular sclerosis, lymphocyte depleted, and lymphocyte predominant. The interval between diagnosis of CLL and subsequent development of HL was about 4.5 years (ranged from 0 to 17.7 years). Before transformation, they were most commonly treated with chlorambucil, cyclophosphamide, and/or fludarabine [4, 5, 13].

The most effective chemotherapy regimen for CLL transformation has not been established. Reported patients most frequently have received combination chemotherapy targeted at HL, including MOPP, ABVD, and CVPP with or without radiotherapy. In some cases, other aggressive cytotoxic regimens with or without rituximab were used. Generally, disease response and clinical outcomes were worse than in de novo HL. The overall response rate was <50 %, and the overall survival of patients was short (in one analyzed cohort was approximately 0.8 year, and in another 1.7 years) [4, 5, 13]. In presented patients, we observed a progressive course of HL. Even very active therapy (DHAP, IVE, autoHSCT) could not change the history of the disease. The effects after conventional therapy are still unsatisfactory, and there is an immense need for new therapeutic approaches. The presence of EBV in most cases of HL, transformed from CLL, represents a potential “tumor-specific” targeting opportunity. Nucleoside-type antiviral agents are ineffective in malignancies associated with latent EBV because the viral enzyme target of these antiviral drugs, thymidine kinase (TK), is not expressed. Therefore, a potential drug should induce EBV-TK expression in latently infected B cells and sensitize EBV-positive lymphoma cells to apoptosis induced by ganciclovir [17]. Such a drug is the pan-histone deacetylase (pan-HDAC) inhibitor arginine butyrate (or other HDAC inhibitors) which with the mentioned above ganciclovir appeared to have significant biologic activity in vivo in EBV-associated lymphomas, which are refractory to other regimens [10, 18]. The other therapeutic option could be monitoring of EBV viral load during fludarabine treatment of CLL and its lowering using antiviral therapy. There is an open question whether such strategy would be useful for preventing CLL transformation into an EBV-associated lymphoma [13]. A number of promising new drugs in HL are currently being evaluated in clinical trials and might further improve the treatment of HL. Among them, there are monoclonal antibodies targeting CD30, whose strong expression is observed on H–RS cells. Recently, a new antibody drug conjugate, brentuximab vedotin, demonstrated very good efficacy and tolerability in a phase I study [19]. Currently, brentuximab vedotin is registered for the treatment of relapsed and refractory HL, not for the treatment of HL transformed from CLL. However, in future, it may become a therapeutic option also in this group of patients.
